# Dental Anxiety Among Undergraduate Dental Students: A Comparative Analysis of MK-DAS and MDAS

**DOI:** 10.3390/healthcare14131920

**Published:** 2026-07-01

**Authors:** Musa Kazim Ucuncu, Merve Yildirim Ucuncu, Zeynep Hale Keles

**Affiliations:** 1 Department of Restorative Dentistry, Hamidiye Faculty of Dental Medicine, Health Sciences University, Istanbul 34668, Türkiye; ucuncumusakazim@gmail.com; 2 Istanbul Provincial Directorate of Health Okmeydani Oral and Dental Health Center, Istanbul 34384, Türkiye; merveyildirim2604@gmail.com; 3 Department of Restorative Dentistry, Faculty of Dentistry, Atlas University, Istanbul 34406, Türkiye

**Keywords:** dental anxiety, MDAS, MK-DAS, survey

## Abstract

**Background/Objectives:** This study included first- to fifth-year dental students and aimed to compare dental anxiety levels using the Musa Kazım Dental Anxiety Scale (MK-DAS) and the Modified Dental Anxiety Scale (MDAS). **Methods:** A cross-sectional, web-based study was conducted at Altınbaş University Faculty of Dentistry (20–31 May 2024). A total of 200 students (40 per academic year) completed the 7-item MK-DAS (cut-off ≥ 17) and the 5-item MDAS (cut-off ≥ 15), and toothbrushing frequency was recorded. Between-year differences were assessed using Kruskal–Wallis tests with effect sizes (ε^2^). The association between academic year and anxiety scores was further evaluated using multivariable linear regression adjusted for age and gender. Concordance between scales was assessed using Spearman’s correlation. Statistical significance was set at α = 0.05. **Results:** Participants were 62.5% female (n = 125), with a mean age of 22.22 ± 2.08 years; 75.5% reported brushing twice daily. Anxiety scores differed significantly across academic years (MDAS: H = 39.05, ε^2^ = 0.184, *p* < 0.001; MK-DAS: H = 43.07, ε^2^ = 0.200, *p* < 0.001). Second-year students had the highest mean scores (MDAS: 14.08 ± 4.53; MK-DAS: 18.40 ± 4.83), whereas fourth-year students had the lowest MDAS scores (8.43 ± 3.37). High anxiety prevalence was 23.0% (MDAS) and 29.0% (MK-DAS). The scales were strongly correlated (Spearman’s ρ = 0.784, *p* < 0.001). Gender-related differences were significant only in second-year students. The association between academic year and anxiety scores remained significant after adjustment. **Conclusions:** Dental anxiety demonstrated a non-linear distribution across academic years, peaking in second-year students. MK-DAS showed strong concordance with MDAS. Findings should be interpreted cautiously given the cross-sectional, web-based design.

## 1. Introduction

Anxiety experienced by patients prior to or during dental treatment is characterised as a complex behavioural phenomenon associated with physiological arousal in response to both internal and external stimuli [1]. Dental anxiety, often referred to as odontophobia, is a common condition that can impose constraints on clinicians’ ability to deliver care and give rise to considerable difficulties for patients. Those who suffer from such anxiety not only tend to neglect their oral health but may also avoid seeking treatment altogether [2]. Although dentistry has witnessed therapeutic and preventive advances in recent decades aimed at improving oral health, substantial progress in eliminating patients’ fear of the dentist has yet to be achieved [3].

Particularly in the early months of 2020, the emergence of COVID-19 precipitated the onset of a pandemic that rapidly engulfed the entire world. The ease with which the causative microorganism could be transmitted via airborne droplets, together with its ready bidirectional spread between patients and clinicians, rendered both dental practitioners and the individuals seeking treatment from them especially vulnerable to COVID-19 transmission [4]. Moreover, the high-speed rotary instruments routinely employed during dental procedures generated droplets and aerosols, thereby creating ample opportunity for cross-infection and for transmission to other patients attending the clinic [5]. The oral cavity, by virtue of its distinctive anatomical architecture, the structural complexity of the dentition, its abundant microbiota and its unique secretory milieu, constitutes a particularly conducive environment for infectious disease transmission; even minor deviations from established hygiene protocols may exponentially amplify the risk to both dental professionals and patients [6]. Furthermore, the high transmissibility of COVID-19 during that period fostered considerable apprehension among individuals regarding the possibility of acquiring infection following a medical or dental visit, a concern that in turn promoted avoidance of dental attendance and, in essence, intensified dental anxiety.

Instruments such as the Dental Fear Survey (DFS), the Dental Anxiety Scale (DAS), and the Modified Dental Anxiety Scale (MDAS) have been widely employed to detect dental anxiety [7,8,9]. The literature comprises numerous studies conducted across diverse age cohorts and sociodemographic strata [10,11,12,13,14,15]. Moreover, the original MDAS appears to have been translated into Turkish and subjected to reliability and validity assessments [16]. While the medical literature contains several investigations into fear and anxiety levels during the COVID-19 pandemic [17,18,19], the dentistry literature includes only a single study that quantified dental anxiety in relation to avoidance of dental treatment and reluctance to seek care owing to infection-risk concerns during the pandemic. In that study, a novel instrument—Musa Kazım’s Dental Anxiety Scale (MK-DAS)—was developed in line with contemporary trends and made available to researchers [10].

The MK-DAS yields an overall, comprehensive measure of dental anxiety by explicitly assessing patients’ concerns about the risk of contracting infectious diseases as a consequence of dental visits, and in this respect has been described as a more innovative and contemporary instrument than the MDAS [10]. MK-DAS encompasses not only anxiety related to the perceived risk of acquiring an infection as a consequence of dental clinic visits, but also a range of well-established triggers of dental anxiety (e.g., waiting prior to treatment, the use of injections, and the use of rotary instruments or conventional hand instruments). Accordingly, it has been introduced to the literature as a comprehensive measure of dental anxiety [10]. However, despite the scale having undergone validity and reliability testing, its application for measuring dental anxiety among dental students remains limited. Moreover, studies examining dental anxiety in dental students, students from other disciplines, and patient populations are relatively few and can be regarded as scarce [3,20]. It is plausible to expect that senior students, who have accrued clinical experience, will display different—typically lower—levels of dental anxiety compared with their junior peers [20]. While elevated levels of dental fear are understandable in individuals defined as patients, those who have been trained in dental treatment protocols and attained competence in applying these protocols to patients would be expected to report relatively lower anxiety levels [2]. Above all, the contemporary literature indicates that dental anxiety remains a salient concern among dental students, and that procedure-related triggers continue to be documented in educational settings through the use of diverse dental anxiety scales [14,21]. Moreover, persistent heterogeneity in the psychometric instruments employed to assess dental anxiety has been highlighted, underscoring the need for transparent comparative studies that evaluate newer measures against established benchmarks and report within-sample concordance across scales [21].

In view of the foregoing, the objective of our study is to conduct a comparative assessment of dental anxiety levels among dental students from first through fifth year by administering the novel, contemporary MK-DAS alongside the MDAS, and to evaluate the correlation between the two instruments. The study hypotheses are: (1) the two scales will demonstrate a positive correlation; and (2) dental anxiety scores on both measures will exhibit a declining trend with advancement from first-year to fifth-year students.

## 2. Materials and Methods

Ethics approval was sought from the Altinbas University Health Sciences Scientific Research Ethics Committee, and the study was initiated following receipt of approval (2024/9). This cross-sectional observational study was conducted and reported in accordance with the Strengthening the Reporting of Observational Studies in Epidemiology (STROBE) Statement for cross-sectional studies, and the completed STROBE checklist is provided as a supplementary file.

G*Power software (G*Power version 3.1.9.6, , Heinrich Heine University, Düsseldorf, Germany) was used to conduct the power analysis. For the comparison of dental anxiety levels across first- through fifth-year students, an effect size corresponding to Cohen’s convention for a medium effect (f = 0.25) was assumed. Statistical significance was set at α = 0.05 and the target study power at 1 − β = 0.80. Sample sizes were planned to be equal across groups, and under these assumptions a sample of 40 participants per year was targeted (n = 40 each per class; N = 200).

The study was conducted online at Altınbaş University Faculty of Dentistry from 20 to 31 May 2024. Undergraduate dental students from first through fifth year were included. The survey was administered to participants via Google Forms. Participants were permitted to proceed only after reading and affirming an informed voluntary consent form. Additionally, respondents were asked whether they had any diagnosed psychological disorder and whether they had been receiving regular psychotropic medication during the preceding six months; individuals who answered “yes” to either question were excluded from the study.

The survey comprised three sections. In the first section, participants were presented with sociodemographic questions and asked to indicate their age, gender, and the number of times they brush their teeth per day. In the second section, respondents answered the MK-DAS items. In the third section, the MDAS was completed. The MK-DAS is a seven-item instrument; the items were as follows: (1) “How do you feel when you are en route to your appointment?”, (2)” How do you feel while waiting in the clinic waiting room for your dentist to call you in?”, (3) “After entering the treatment room, how concerned are you about whether the surfaces of any devices and materials expected to be used during the procedure are clean and sterile?”, (4) “Immediately prior to the start of treatment, you observe for the first time the “needle” (injection) in your clinician’s hand. How do you feel?”, (5) “How do you feel when the clinician is working in your mouth with noisy instruments and rotating devices?”, (6) “How do you feel when the clinician is working in your mouth with quiet hand instruments?”, (7) “In the clinical environment or during treatment, how do you feel about the possibility of contracting infectious diseases such as COVID-19, hepatitis B, or influenza?”. The MK-DAS was administered using a Likert-type response format with options scored from 1 to 5: 1 = “no anxiety/I feel relaxed”, 2 = “I feel somewhat uneasy”, 3 = “I feel tense”, 4 = “I feel anxious/distressed”, and 5 = “I experience intense fear”. The total score ranges from a minimum of 7 to a maximum of 35. A cut-off value of ≥17 has been established to denote high anxiety [10].

The third section of the questionnaire comprised the MDAS to assess participants’ dental anxiety. The MDAS items were as follows: (1) “How would you feel if you were to visit the dentist tomorrow?”, (2) “How would you feel while waiting in the waiting room for your turn?”, (3) “How would you feel if a drilling instrument were being used on your tooth?”, (4) “How would you feel if your dental calculus were being scaled/cleaned?”, (5) “How would you feel if a needle were to be used inside your mouth?”. The MDAS employs a five-point Likert response format, with responses anchored as: 1 = “Not anxious”, 2 = “Slightly anxious”, 3 = “Anxious”, 4 = “Very anxious”, and 5 = “Extremely anxious”. Total scores range from a minimum of 5 to a maximum of 25. The MDAS has demonstrated validity and reliability, and a cut-off score of ≥15 has been established to indicate high dental anxiety [16,22].

### Statistical Analysis

Statistical analyses were performed using IBM SPSS Statistics (version 31.0.0, IBM Corp., Armonk, NY, USA). Descriptive statistics (mean, standard deviation, median, interquartile range [Q1–Q3], and minimum–maximum) were calculated. Responses obtained from the Likert-type scales were summed to yield total MDAS and MK-DAS scores, which were compared across academic years (first through fifth). Normality was assessed using the Shapiro–Wilk test and by inspection of skewness and kurtosis values; homogeneity of variances was evaluated with Levene’s test. For comparisons across the five independent year groups the nonparametric Kruskal–Wallis H test was applied, with subsequent pairwise comparisons conducted using Mann–Whitney U tests with Holm–Bonferroni correction to control for multiple testing. To evaluate the trend in anxiety scores across ascending academic years, Spearman’s rank correlation (Spearman’s rho) was employed. The association between the two scales (MDAS and MK-DAS) was also examined using Spearman’s rho. To further examine whether differences in anxiety scores across academic years persisted after adjustment for potential confounders, multivariable linear regression analyses were performed with MDAS and MK-DAS total scores as dependent variables and academic year, gender, and age as independent variables. Prior to interpreting the multivariable linear regression models, multicollinearity among independent variables was assessed using tolerance and variance inflation factors (VIF). Because age and academic year are inherently related in student cohorts, collinearity diagnostics were inspected to ensure stable estimation. The statistical significance was set at α = 0.05.

## 3. Results

### 3.1. Participant Characteristics and Oral Hygiene Habits

Sociodemographic and general characteristics of all participants are presented in Table 1. Of the 200 participants, 62.5% were female (n = 125) and 37.5% were male (n = 75). The mean age of the sample was 22.22 ± 2.08 years. Overall, the predominant brushing frequency was twice daily (n = 151, 75.5%). After excluding participants who reported irregular brushing, the highest mean brushing frequency by academic year was observed among fourth-year students (2.00 ± 0.00), whereas the lowest was observed in the second-year students (1.72 ± 0.45).

### 3.2. Dental Anxiety Scores Across Academic Years

**Figure 1:**
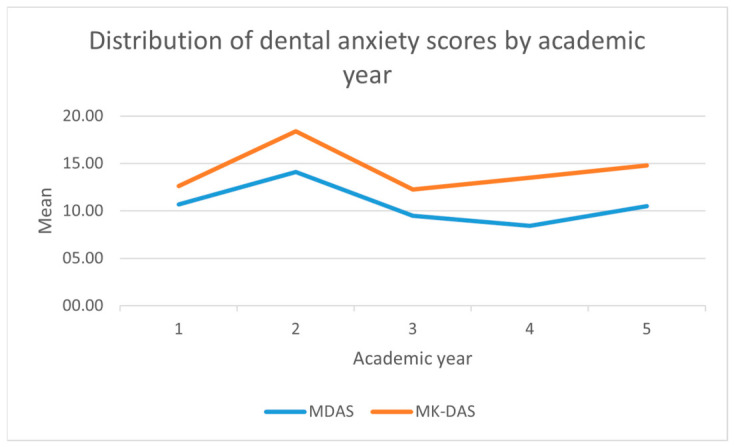
Illustration of the variation in MDAS and MK-DAS values across academic years.

Dental anxiety scores differed significantly across academic years on both instruments (MDAS: H = 39.05, ε^2^ = 0.184, *p* < 0.05; MK-DAS: H = 43.07, ε^2^ = 0.200, *p* < 0.05). With respect to the MDAS, second-year students exhibited the highest anxiety (14.08 ± 4.53), while fourth-year students demonstrated the lowest levels (8.43 ± 3.37) (Table 2). Similarly, MK-DAS scores were highest among second-year students (18.40 ± 4.83), whereas third-year students showed a comparatively lower anxiety level (12.25 ± 4.31) (Table 2) (Figure 1).

### 3.3. Pairwise Comparisons and Effect Sizes

Post-hoc pairwise comparisons indicated that second-year students had significantly higher anxiety scores than the other class years on both instruments (*p* < 0.05). For MDAS, fourth-year students exhibited significantly lower scores than fifth-year students, whereas there was no statistically significant difference between first- and third-year students (*p* > 0.05). Regarding MK-DAS, scores were significantly lower in third-year than in fifth-year students, while the comparison between first- and fourth-year students did not reach statistical significance (*p* > 0.05). When examined in terms of effect sizes, the values were of a moderate-to-large magnitude (ε^2^ ≈ 0.18–0.20), indicating a meaningful differentiation in dental anxiety scores.

### 3.4. Trend Analysis and High Dental Anxiety Prevalence

A small but statistically significant negative trend in MDAS scores across academic years (first through fifth) was observed (Spearman’s rho = −0.172, *p* = 0.015). No significant monotonic trend was detected for the MK-DAS (Spearman’s rho = 0.028, *p* = 0.692) (Table 3). According to the established cut-offs, 46 participants (23%) were classified as having high dental anxiety by the MDAS, whereas 58 participants (29%) met the high-anxiety criterion on the MK-DAS. The greatest prevalence of high anxiety was observed among second-year students, while the lowest prevalence was found in fourth-year students (Table 3). A strong, positive association between the two instruments was identified (Spearman’s rho = 0.784, *p* < 0.001) (Table 2). Pairwise comparisons indicated that second-year students exhibited significantly higher dental anxiety than all other years on both the MDAS and the MK-DAS (*p* < 0.005). Additionally, MDAS scores differed significantly between fourth- and fifth-year students, whereas MK-DAS scores differed significantly between third- and fifth-year students (*p* < 0.005). When stratified by gender within academic year, statistically significant differences on both the MDAS and MK-DAS were observed only in the second-year students (*p* = 0.022 for MDAS; *p* = 0.004 for MK-DAS). No significant gender-related differences in dental anxiety were detected in the other years (*p* > 0.05). Changes in dental anxiety according to brushing frequency and gender are presented in Table 4. Also, multivariable linear regression analyses adjusted for age and gender showed that academic year remained significantly associated with both MDAS and MK-DAS scores (MDAS: overall *p* < 0.001; MK-DAS: overall *p* < 0.001). In the adjusted model, second-year students had significantly higher anxiety scores than first-year students on both the MDAS (β = 3.62, *p* < 0.001) and the MK-DAS (β = 5.86, *p* < 0.001) (Table 5). Also, internal consistency was high for MDAS (Cronbach’s α = 0.899) and acceptable for MK-DAS (Cronbach’s α = 0.792). VIF values ranged from 1.09 to 2.86 and tolerance values ranged from 0.35 to 0.92, indicating no problematic multicollinearity in the fitted models (Table 6); although age and academic year were moderately correlated (Pearson’s r = 0.70), the predictors did not show excessive overlap and coefficient estimates were considered stable.

## 4. Discussion

Based on the obtained results, the study hypotheses were only partially upheld. The first hypothesis—that the two instruments would exhibit a positive correlation—was corroborated. Conversely, the second hypothesis—that dental anxiety scores would demonstrate a progressive decline with advancement from lower to higher academic years—was not substantiated.

To date, a wide variety of dental anxiety scales have been employed in the literature. One of the earliest and most widely used instruments developed to identify dental anxiety is Corah’s Dental Anxiety Scale, which has achieved broad applicability owing to its brevity and practical utility [8]. However, because it did not include an item assessing fear of injection, it was subsequently modified by Humphris, who incorporated this component into the structure and thereby developed a five-item revised version, termed the Modified Dental Anxiety Scale (MDAS) [9]. The Dental Fear Survey (DFS), by contrast, provides a more comprehensive assessment of dental anxiety, evaluating avoidance behaviour, physiological arousal and fear responses to specific dental stimuli in greater detail, and is therefore particularly well suited to psychometric investigation [7]. The Index of Dental Anxiety and Fear (IDAF-4C+), which may be regarded as a comparatively more recent instrument, offers a multidimensional assessment of dental anxiety across cognitive, behavioural, emotional and physiological domains, while also addressing avoidance of dental treatment through its supplementary modules [23]. According to recent studies, none of the scales used to assess dental anxiety can be regarded as a definitive and indisputable gold standard. Rather, the choice of instrument is likely to vary depending on the population under investigation and the particular dimension of anxiety intended to be measured [24]. The MDAS remains among the most widely used instruments, largely owing to its brevity and practical utility relative to other scales. Nevertheless, heterogeneity in its administration and interpretation across studies has also been documented, which may compromise comparability between findings [25]. Furthermore, bibliometric evidence suggests that dental anxiety research is becoming increasingly context-sensitive and is being approached within a more multidimensional conceptual framework [26]. In summary, the concurrent use of both the MDAS and the MK-DAS in the present study was intended to enable cross-scale comparison and to evaluate the degree of concordance between a well-established, widely used, easy-to-administer instrument that does not impose substantial respondent burden, and a more recently developed contemporary scale.

While a variety of instruments exist to quantify dental anxiety, the Modified Dental Anxiety Scale (MDAS)—one of the most frequently employed measures—was derived from the original DAS by the addition of a fifth item specifically addressing anxiety provoked by the prospect of injection [9,27]. The MDAS has been used across studies with differing cut-off thresholds: Caltabiano et al. adopted a relatively high threshold, setting the cut-off at 19 [12], whereas Storjord et al. characterised individuals scoring within a certain intermediate range as having moderate dental anxiety and designated scores of 18 and above as indicative of high dental anxiety [28]. To provide a theoretical foundation for the instrument’s content and structure, Humphris and Newton reported evidence supporting a two-factor solution, suggesting that the scale may be meaningfully partitioned into “anticipatory” and “treatment-related” subscales [27].

With the advent of the COVID-19 pandemic, shifting perceptions and behaviours regarding infectious diseases precipitated patient avoidance of care and disruptions within health services [29,30,31]. However, the extent to which these developments translated into heightened dental anxiety remained unclear, and no instrument specifically designed to capture anxiety about infection risk in dental settings was available. In response, the MK-DAS was developed to quantify the anxiety individuals might experience concerning the risk of contracting infectious diseases (e.g., COVID-19, HIV, influenza) as a consequence of attending dental clinics. Originally conceived as an eight-item instrument, factor analyses led to the removal of the eighth item, yielding a two-factor structure. Apart from the scale’s reliability and validity study [10], no published research applying the MK-DAS to independent populations has been identified; accordingly, the present investigation represents the first cross-sectional study to incorporate the MK-DAS.

Recent intervention-based evidence also underscores the potential role of non-pharmacological strategies in the management of dental anxiety. In a triple-blind randomized clinical trial, Argueta-Figueroa et al. reported that lavender essential oil aromatherapy did not produce a statistically significant reduction in self-reported MDAS scores compared with placebo; however, it was associated with lower salivary cortisol levels, heart rate, respiratory rate, and local anesthetic requirements among patients undergoing mandibular third molar surgery. Although the design and study population of that trial differ from those of the present cross-sectional study, its findings highlight the importance of evaluating dental anxiety not solely through self-report instruments, but also in relation to physiological responses and contextual factors [32].

Advancement through the curriculum is associated with greater dental knowledge, heightened awareness, and a consequent reduction in dental anxiety; indeed, dental students may exhibit lower levels of dental anxiety than their counterparts in other health disciplines [2,20,28,33]. Our findings indicate that anxiety scores across academic years do not follow a uniform linear trajectory; rather, they exhibit a non-linear pattern characterized by partial decreases between certain years and partial increases between others. However, it is noteworthy that both instruments reveal an initial increase in anxiety from first to second year, followed by a pronounced decline and a modest rebound by fifth year. The salient observation is the markedly elevated anxiety among second-year students on both scales. One possible explanation for the higher anxiety scores observed among second-year students may lie in the transitional nature of this phase of dental education in Türkiye. In many dental curricula, the second year is characterised by intensified preclinical instruction and simulation-based training, whereas direct patient contact remains limited. Although the present study did not directly examine the mechanisms underlying this pattern, it is conceivable that restricted clinical exposure, together with an increased academic workload, may contribute to elevated anxiety during this period. By contrast, greater participation in clinical procedures in the later years may facilitate adaptation through repeated exposure and growing familiarity, thereby attenuating anxiety. Nevertheless, these interpretations should be approached with caution and warrant confirmation through longitudinal studies specifically designed to investigate these factors. Moreover, a heavier theoretical and academic workload in the second year relative to the first may elevate general anxiety, thereby potentiating dental-specific fears. Conversely, repeated exposure to simple procedures—such as administration of local anaesthesia—and active participation in their delivery can foster acclimatisation and promote adaptive coping strategies based on personal clinical experience [34,35]. It is precisely this process of adaptation that appears to be incompletely developed in the second-year students. Moreover, the association between academic year and anxiety scores remained significant after adjustment for age and gender, suggesting that the observed pattern cannot be explained solely by differences in these demographic characteristics.

Individuals who prioritise oral health and possess heightened oral-hygiene awareness are not expected to exhibit elevated levels of dental anxiety [3]. In our study, the superior tooth-brushing habits and concomitantly lower anxiety levels observed among fourth-year students are consistent with this body of literature. Fourth-year students appear to have overcome earlier limitations in clinical exposure and adaptation. By this stage their clinical experience and patient interactions have markedly increased, a pattern that plausibly facilitated habituation and thereby contributed to reduced dental anxiety. The relatively lower anxiety observed in third- and fourth-year students compared with second-year students can thus be ascribed to accrued clinical exposure and consequent acclimatisation. Conversely, fifth-year students face distinct stressors—such as imminent graduation, preparation for specialty examinations, and the completion of graduation-related academic requirements—which are commonly reported sources of distress [36]. Broader concerns about the future and elevated levels of general anxiety or depressive symptomatology in the pre-graduation period may, in turn, exert a modest exacerbating influence on dental-specific anxiety [37].

If the limitations of the study are to be enumerated, they include the following. First, administering the questionnaire online rather than in the clinical setting may have influenced responses by removing contextual cues inherent to the dental environment; consequently, ecological validity and situational provocation of anxiety were attenuated. Second, a larger sample drawn from a broader population would increase statistical power and improve the precision with which trends across academic years in dental anxiety can be estimated and generalized. Third, although a recent meta-analysis recommended interpreting MDAS results in intervals/ranges rather than relying solely on a single cut-off score, this study classified high dental anxiety on the basis of a single threshold [38], which may reduce comparability with interval-based approaches and obscure gradations of clinical relevance. Fourth, the cross-sectional design provides only a snapshot of students’ dental anxiety at the time of data collection; it cannot establish how individual anxiety trajectories evolve as students progress through the curriculum. Accordingly, future research should prioritise larger, multi-centre samples and employ longitudinal or prospective designs to more robustly assess temporal changes and causal factors underlying shifts in dental anxiety across academic years. In addition, although excluding participants with self-reported psychological disorders and/or medication use reduced potential confounding, it may have introduced selection bias and limited the generalizability of the findings to the broader student population. In addition, although excluding participants with self-reported psychological disorders and/or medication use reduced potential confounding, it may have introduced selection bias and limited the generalizability of the findings to the broader student population. Moreover, the online mode of data collection may have preferentially included students who were more willing or available to participate, thereby introducing a potential self-selection bias. Therefore, while the observed differences across academic years are supported by the present data, explanations related to curriculum structure, clinical exposure, workload, or pre-graduation stress should be interpreted as plausible hypotheses rather than definitive causal mechanisms.

## 5. Conclusions

Dental anxiety levels do not exhibit a uniform pattern of change across academic years; rather, they increase between certain years and decrease between others. In this study second-year dental students were found to have relatively higher dental anxiety compared with other years. Moreover, daily tooth-brushing frequency is inversely related to dental anxiety levels. Both the MDAS and the contemporary, novel MK-DAS serve researchers as two similar dental anxiety scales that demonstrate a strong, positive correlation with one another. These findings may provide implications for dental education curricula by highlighting the importance of structured clinical exposure and targeted support, particularly during academic years associated with higher levels of anxiety. Furthermore, student well-being interventions and further validation studies in larger and more diverse populations are warranted to strengthen the applicability and clinical interpretability of the MK-DAS.

## Figures and Tables

**Table 1:** Overall data summary for all participants.

	General	First-Year	Second-Year	Third-Year	Fourth-Year	Fifth-Year
AgeMean ± std;Median(Min–Max)	22.22 ± 2.08;22.0(19–36)	20.20 ± 0.76; 20.0(19–22)	21.05 ± 0.88; 21.0(19–23)	22.32 ± 2.59; 22.0(20–36)	23.30 ± 1.30; 23.0(22–29)	24.23 ± 1.17; 24.0(22–27)
Female (n, %)	125 (62.5%)	28 (70.0%)	34 (85.0%)	21 (52.5%)	24 (60.0%)	18 (45.0%)
Male (n, %)	75 (37.5%)	12 (30.0%)	6 (15.0%)	19 (47.5%)	16 (40.0%)	22 (55.0%)
Once a day (n, %)	35 (17.5%)	12 (30.0%)	10 (25.0%)	9 (22.5%)	0 (0.0%)	4 (10.0%)
Twice a day (n, %)	151 (75.5%)	24 (60.0%)	26 (65.0%)	31 (77.5%)	40 (100.0%)	30 (75.0%)
Three times a day (n, %)	5 (2.5%)	4 (10.0%)	0 (0.0%)	0 (0.0%)	0 (0.0%)	1 (2.5%)
Irregular (n, %)	9 (4.5%)	0 (0.0%)	4 (10.0%)	0 (0.0%)	0 (0.0%)	5 (12.5%)
Frequency of toothbrushing * Mean ± std;Median(Min–Max)	1.84 ± 0.43; 2.0 (1–3)	1.80 ± 0.61; 2.0 (1–3)	1.72 ± 0.45; 2.0 (1–2)	1.77 ± 0.42; 2.0 (1–2)	2.00 ± 0.00; 2.0 (2–2)	1.91 ± 0.37; 2.0 (1–3)

* without irregular brushing.

**Table 2:** Mean scores for each class according to the dental anxiety scales and correlation analysis between the scales.

	MDAS * Mean + sd.	MDAS Median (Q1–Q3)	MK-DAS * Mean + sd.	MK-DAS Median (Q1–Q3)	Correlation Analysis Between the Scales
First-year	10.70 ± 4.30 ^ab^	10 (7–14.25)	12.60 ± 4.44 ^ab^	11 (9.75–15)	ρ = 0.784, *p* < 0.001 **
Second-year	14.08 ± 4.53 ^c^	14 (11–17)	18.40 ± 4.83 ^d^	18 (14.75–20)	
Third-year	9.05 ± 4.00 ^ab^	8 (6–10.25)	12.25 ± 4.31 ^a^	11 (9–14)	
Fourth-year	8.43 ± 3.37 ^a^	8 (6–9)	13.50 ± 4.03 ^ab^	12.5 (11–15)	
Fifth-year	10.48 ± 3.32 ^b^	10 (8–14.25)	14.80 ± 3.92 ^bc^	14 (12–18)	

* Kruskal–Wallis test followed by pairwise Mann–Whitney U tests with Holm–Bonferroni correction for multiple comparisons. Different superscript letters indicate statistically significant differences between classes, *p* < 0.005. ** Spearman’s rank correlation.

**Table 3:** Identification of participants with high dental anxiety and assessment of the trend in dental anxiety across classes.

Class	MDAS ≥ 15 (n, %)	MDAS *	MK-DAS ≥ 17 (n, %)	MK-DAS *
1	10 (25%)	ρ = −0.172, *p* < 0.05	6 (15%)	ρ = 0.028, *p* = 0.692
2	19 (47.5%)		27 (67.5%)	
3	5 (12.5%)		7 (17.5%)	
4	2 (5%)		5 (12.5%)	
5	10 (25%)		13 (32.5%)	

* Spearman’s rank correlation. *p* < 0.05.

**Table 4:** For each class, *p*-values for gender- and toothbrushing-frequency comparisons are reported.

Class	MDAS (Gender)	MK-DAS (Gender)	MDAS (Frequency of Toothbrushing)	MK-DAS (Frequency of Toothbrushing)
1	0.449 ^U^	0.320 ^U^	0.048 ^KW^*	0.008 ^KW^*
2	0.026 ^t^*	0.004 ^U^*	0.163 ^KW^	0.116 ^KW^
3	0.529 ^U^	0.313 ^U^	0.413 ^KW^	0.858 ^KW^
4	0.124 ^U^	0.454 ^U^	------------	---------------
5	0.133 ^U^	0.183 ^U^	0.105 ^KW^	0.003 ^KW^*

U: Mann–Whitney U test. t: Welch’s *t*-test. KW: Kruskal–Wallis Test. * *p* < 0.05.

**Table 5:** Multivariable linear regression models for MDAS and MK-DAS total scores adjusted for academic year, gender, and age.

Variable	MDAS β (95% CI)	*p*	MK-DAS Β (95% CI)	*p*
2nd year vs. 1st year	3.62 (1.86 to 5.39)	<0.001	5.86 (3.93 to 7.79)	<0.001
3rd year vs. 1st year	−0.94 (−2.85 to 0.98)	0.337	0.47 (−1.62 to 2.56)	0.659
4th year vs. 1st year	−1.26 (−3.34 to 0.83)	0.235	1.91 (−0.37 to 4.18)	0.100
5th year vs. 1st year	1.11 (−1.20 to 3.43)	0.343	3.65 (1.13 to 6.18)	0.005
Male vs. female	−0.17 (−1.35 to 1.01)	0.779	−1.21 (−2.50 to 0.08)	0.066
Age	−0.32 (−0.70 to 0.05)	0.089	−0.29 (−0.69 to 0.12)	0.168

MDAS: R^2^ = 0.215, adjusted R^2^ = 0.191, *p* < 0.001; MK-DAS: R^2^ = 0.235, adjusted R^2^ = 0.211. *p* < 0.001. β coefficients were obtained from multivariable linear regression models including academic year, gender, and age. First-year and female students were treated as the reference categories. CI: confidence interval.

**Table 6:** Multicollinearity diagnostics for the multivariable linear regression models (VIF and tolerance).

Predictor	VIF	Tolerance (=1/VIF)
5th year (5th year & 1st year)	2.86	0.35
4th year	2.32	0.43
Age	1.99	0.50
3rd year	1.96	0.51
2nd year	1.67	0.60
Male & Female	1.09	0.92

## Data Availability

The raw data supporting the conclusions of this article will be made available by the authors on request.

## Supplementary Materials

The following supporting information can be downloaded at: https://www.mdpi.com/article/10.3390/healthcare14131920/s1, Survey S1: MK-DAS; Survey S2: MDAS (The surveys are provided in Turkish).
