# Supplementary material for: Dental Anxiety Among Undergraduate Dental Students: A Comparative Analysis of MK-DAS and MDAS
Source: Healthcare (Basel). 2026 Jul 1;14(13):1920. doi: 10.3390/healthcare14131920 (PMC13361449; doi:10.3390/healthcare14131920)
Supplement: Supplementary file 1 [file healthcare-14-01920-s001.zip › healthcare-4343403-supplementary.pdf]

MK-DAS

1)Randevunuz için yola çıktığınızda nasıl hissedersiniz?
Tedavi zamanım yanaştığı için mutlu olurum.
Herhangi bir endişe duymam.
Gerginlik hissederim.
Kaygılı ve sıkıntılı hissederim.
Çok büyük bir korkuya kapılırım

2)Klinikte bekleme odasında hekiminizin sizi odaya almasını bekliyorsunuz? Nasıl hissedersiniz?
Herhangi bir korku duymam, rahat olurum
Bir miktar huzursuz olurum.
Gerginlik hissederim
Kaygılı ve sıkıntılı hissederim.
Çok büyük bir korkuya kapılırım

3)Odaya girdikten sonra işlem esnasında kullanılacağını düşündüğünüz her türlü cihaz ve materyal yüzeylerinin temiz ve steril olup olmaması konusunda nasıl hissedersiniz?
Herhangi bir korku duymam, rahat olurum
Bir miktar huzursuz olurum.
Gerginlik hissederim
Kaygılı ve sıkıntılı hissederim.
Çok büyük bir korkuya kapılırım

4)Tedavi başlamadan hemen önce hekiminizin elinde ilk kez "iğne" (enjeksiyon)'yi gördünüz. Nasıl hissedersiniz?
Herhangi bir korku duymam, rahat olurum
Bir miktar huzursuz olurum.
Gerginlik hissederim
Korkarım
Çok büyük bir korkuya kapılırım

5)Hekim ağzınız içinde gürültü çıkaran her türlü alet ve dönen enstrümanlarla çalışırken nasıl hissedersiniz?
Herhangi bir korku duymam, rahat olurum
Bir miktar huzursuz olurum.
Gerginlik hissederim
Kaygılı ve sıkıntılı hissederim.
Çok büyük bir korkuya kapılırım

6)Hekim ağzınız içinde sessiz el aletleriyle çalışırken nasıl hissedersiniz?
Herhangi bir korku duymam, rahat olurum
Bir miktar huzursuz olurum.
Gerginlik hissederim
Kaygılı ve sıkıntılı hissederim.
Çok büyük bir korkuya kapılırım

7)Klinik ortamında veya tedavi esnasında COVID-19, Hepatit B, Influenza vb. bulaşıcı hastalık kapma ihtimaline karşı nasıl hissedersiniz?
Herhangi bir korku duymam, rahat olurum
Bir miktar huzursuz olurum.
Gerginlik hissederim
Kaygılı ve sıkıntılı hissederim.
Çok büyük bir korkuya kapılırım

MDAS

1.Yarın diş hekimine gidecek olsanız kendiniz nasıl hissedersiniz?

Eğlenceli bir deneyim olacağını düşünürüm
Bu durumu hiç önemsemem ve hiç endişe etmem
Çok az huzursuzluk duyarım
Hoş olmayan tatsız(ağrılı) bir olay olacağını düşündüğüm için korkarım
Diş hekimi ne yapacak diye korkarım.

2.Diş hekimi muayenehanesindesiniz ve sıranın size gelmesini bekliyorsunuz. Kendinizi nasıl hissedersiniz?

Rahat hissederim,
Biraz huzursuz hissederim
Gergin hissederim
Endişeli ve sıkıntılı hissederim.
Çok korkarım , vücudumda terleme ve bulantı gibi değişiklikler hissederim.

3.Diş hekiminin koltuğuna oturdunuz ve doktorunuzun tedavi için dönen aletlerini hazırlamasını bekliyorsunuz.Kendinizi nasıl hissedersiniz?

Rahat hissederim,
Biraz huzursuz hissederim
Gergin hissederim
Endişeli ve sıkıntılı hissederim.
Çok korkarım , vücudumda terleme ve bulantı gibi değişiklikler hissederim.

4.Diş hekiminin kotuğuna oturdunuz ve doktorounuz dişetleriniz etrafındaki diş taşlarını temizlemek için kazıyıcı aletlerini hazırlamasını bekliyorsunuz. Kendinizi nasıl hissedersiniz?

Rahat hissederim,
Biraz huzursuz hissederim
Gergin hissederim
Endişeli ve sıkıntılı hissederim.
Çok korkarım , vücudumda terleme ve bulantı gibi değişiklikler hissederim.

5.Diş hekiminiz üst arka dişinizin üstünde dişetinize lokal anestezi enjeksiyonu yapacak olsa kendinizi nasıl hissedersiniz?

Rahat hissederim,
Biraz huzursuz hissederim
Gergin hissederim
Endişeli ve sıkıntılı hissederim.
Çok korkarım , vücudumda terleme ve bulantı gibi değişiklikler hissederim.
